# Osteoinductive micro-nano guided bone regeneration membrane for in situ bone defect repair

**DOI:** 10.1186/s13287-024-03745-w

**Published:** 2024-05-07

**Authors:** Bingqian Wang, Xinfang Xie, Wenbin Jiang, Yichen Zhan, Yifan Zhang, Yaqi Guo, Zhenxing Wang, Nengqiang Guo, Ke Guo, Jiaming Sun

**Affiliations:** 1 Department of Plastic Surgery, Union Hospital, Tongji Medical College, Huazhong University of Science and Technology, Wuhan, 430022 China; 2 Wuhan Clinical Research Center for Superficial Organ Reconstruction, Wuhan, 430022 China

**Keywords:** Metal–organic framework, Biomimetic mineralization, Electrostatic spinning, Macrophage phenotype, Bone regeneration

## Abstract

**Background:**

Biomaterials used in bone tissue engineering must fulfill the requirements of osteoconduction, osteoinduction, and osseointegration. However, biomaterials with good osteoconductive properties face several challenges, including inadequate vascularization, limited osteoinduction and barrier ability, as well as the potential to trigger immune and inflammatory responses. Therefore, there is an urgent need to develop guided bone regeneration membranes as a crucial component of tissue engineering strategies for repairing bone defects.

**Methods:**

The mZIF-8/PLA membrane was prepared using electrospinning technology and simulated body fluid external mineralization method. Its ability to induce biomimetic mineralization was evaluated through TEM, EDS, XRD, FT-IR, zeta potential, and wettability techniques. The biocompatibility, osteoinduction properties, and osteo-immunomodulatory effects of the mZIF-8/PLA membrane were comprehensively evaluated by examining cell behaviors of surface-seeded BMSCs and macrophages, as well as the regulation of cellular genes and protein levels using PCR and WB. In vivo, the mZIF-8/PLA membrane’s potential to promote bone regeneration and angiogenesis was assessed through Micro-CT and immunohistochemical staining.

**Results:**

The mineralized deposition enhances hydrophilicity and cell compatibility of mZIF-8/PLA membrane. mZIF-8/PLA membrane promotes up-regulation of osteogenesis and angiogenesis related factors in BMSCs. Moreover, it induces the polarization of macrophages towards the M2 phenotype and modulates the local immune microenvironment. After 4-weeks of implantation, the mZIF-8/PLA membrane successfully bridges critical bone defects and almost completely repairs the defect area after 12-weeks, while significantly improving the strength and vascularization of new bone.

**Conclusions:**

The mZIF-8/PLA membrane with dual osteoconductive and immunomodulatory abilities could pave new research paths for bone tissue engineering.

## Introduction

Bone defects commonly arise from various causes such as trauma, infections, and tumors [1]. A significant challenge in the healing of large bone defects is the rapid growth of connective tissue that impedes new bone formation. Guided bone regeneration (GBR) technology has been developed to address this issue and is widely used in orthopedics and dentistry [2–3]. The success of GBR treatment hinges on the properties of the barrier membrane, which acts as a physical barrier separating soft tissue from the bone defect area to prevent interference by fibrous connective tissue. By allowing osteoblasts to proliferate within the bone defect area, the GBR membrane facilitates new bone formation. Therefore, the barrier membrane plays a crucial role in promoting the proliferation and regeneration of bone tissue [4]. 

Bone-guided membrane materials used in preclinical applications can be categorized into biodegradable and nonbiodegradable types [5, 6]. The nonbiodegradable bone guide membrane typically consist of materials such as polytetrafluoroethylene [7] and titanium [8, 9]. While these nondegradable membrane offer stable material properties and high mechanical strength, they often require a second surgical procedure of removal, potentially leading to complications like mucosal dehiscence, exposure, and postoperative infection [10]. In order to minimize patient discomfort, costs, tissue invasion, and the risk of new bone tissue loss, biodegradable materials are preferred over nonbiodegradable ones for clinical applications. Therefore, studies focused on absorbable membranes based on polymers (both natural and artificial), including collagen (for example, Bio-Gide™ from Wolhusen, Switzerland) [11], polyglycolic acid [12], polylactic acid (PLA) [13], and polycaprolacton [14]. PLA is a particularly suitable for electrospun membrane preparation due to its biocompatibility and non-bioaccumulative nature in vital organs [15]. However, the lack of osteoinductive properties in PLA presents a significant challenge when utilizing it in bone tissue engineering.

Zeolitic imidazolate framework-8 (ZIF-8), composed of Zn^2+^ and 2-methylimidazolate ligands, has been utilized in biomedical applications owing to its impressive thermal stability, large surface area, rich pore structure, and pH-response characteristics [16]. The degradation and collapse of ZIF-8 have been observed in phosphate-buffered saline (PBS). *Miriam et al.* investigated the degradation mechanism of ZIF-8 degradation in PBS and identified the strong affinity of phosphates for Lewis metal clusters [17]. Consequently, the coordination equilibrium was altered for forming insoluble zinc phosphates, thus enhancing the release of imidazole ligand. Additionally, competitive binding possibly facilitates the exchange of ZIF-8 with anions in different mediums containing abundant inorganic anions and metallic cations [18]. Thus, ZIF-8 has potential to induce biomimetic mineralization in simulated body fluids (SBF), which contain considerable amounts of active metal cations and phosphates. Notably, upon introducing functional metal elements into the implant, a range of immune responses happened in the local microenvironment consequently.

Macrophages can be activated in response to pathophysiological stimuli and polarized into two distinct phenotypes: pro-inflammatory classically activated macrophages (M1 phenotype) and anti-inflammatory alternatively activated macrophages (M2 phenotype). Following bone injury, M1 macrophages play a role in the early stages of inflammation, while M2 macrophages are involved in later bone regeneration processes [19, 20]. M1 macrophages secrete various inflammatory factors such as tumor necrosis factor 𝛼 (TNF𝛼) and interleukin-1𝛽 (IL1𝛽), which contribute to the inflammatory response. Prolonged dominance of M1 macrophages may lead to bone discontinuity and fibrosis [21]. On the other hand, M2 macrophages release anti-inflammatory factors such as interleukin 10 (IL10) and interleukin 4 (IL4), promoting tissue regeneration [22]. A highly efficient and instant transition from M1 to M2 phenotype macrophages is crucial for osteoanagenesis [23, 24]. Previous researches have reported that Ca^2+^ and Zn^2+^ have immunomodulatory properties, and Ca^2+^ served as a promoting factor for the proliferation of osteoblasts [25, 26]. *Zhao et al.* [27] found that Ca–Zn–P coating exhibited satisfactory osteoimmunomodulation ability in following aspects: induction of the M2-phenotype polarization and resulting facilitation of bone formation; modulation of the bone immune microenvironment to facilitate osseointegration *in vivo.* Therefore, ZIF-8 can be confidently considered as an effective inducer of a favorable immune microenvironment after mineralization.

Herein, we propose a novel approach for constructing GBR membranes using electrospinning and technology. The prepared ZIF-8/PLA membrane was anticipated to retain the inherent capability of ZIF-8 to induce biomimetic mineralization. As a result, calcium and phosphorus are deposited on the mineralized ZIF-8/PLA (mZIF-8/PLA), which not only improves the attachment and bone-forming differentiation of BMSCs, but also prompts recruited macrophages to adopt an anti-inflammatory phenotype. These macrophages subsequently secrete anti-inflammatory factors, working together synergistically to enhance bone regeneration.

## Materials and methods

### Synthesis and biomimetic mineralization of ZIF-8

ZIF-8 nanoparticles were synthesized according to a previous procedure with slight asjustmanets [28]. Initially, 3.36 g of zinc nitrate hexahydrate (Sigma, 228,737) was dissolved in 40 ml of solvent (anhydrous methanol) and mixed drop by drop with 8 g of 2-methylimidazole in 80 ml of solvent. The mixture was continuously stirred magnetically for 3.5 h. The resulting ZIF-8 product was then washed twice with a methanol solution, centrifuged at 5000 rpm and 9500 rpm, the supernatant discarded, and finally dried at 70 °C overnight for further purification. Subsequent biomimetic mineralization was carried out using simulated body fluid (SBF), where ZIF-8 was immersed in SBF for 14-day with daily semi-quantitative replacement of SBF solution.

### Characterization of mineralization process of ZIF-8

The EDS elemental mapping experiments together with transmission electron microscopy (TEM) images were conducted on FEI Talos-F200X. Crystalline phases of the mineralized ZIF-8 were characterized by XRD (Nalytical, X’Pert PRO MPD). FT-IR spectra were collected by Nicolet iS10 spectrophotometer (Thermo).

### Preparation of electrospun mZIF-8/PLA membrane

ZIF-8/PLA was fabricated by electrospinning method using a blend of PLA and ZIF-8 (10 wt%). The PLA and mixture ZIF-8/PLA were dissolved in hexafluoroisopropanol at a total concentration of 8% (wt./vol). The electrospinning flow rate is 1.0 mL/h under a high voltage of 22 kV. Fibers were ejected to a rounded rotating collector at a distance of 10 cm. The obtained membranes were then undergoing Freeze drying. For biomimetic mineralization, 40 mL SBF was added to the centrifuge tube containing six immersed membrane samples, followed by a standing period of 7 days at 37℃ with daily SBF change for maintaining identical ionic strength during this process.

### Characterization of mZIF-8/PLA membrane

Scanning electron microscopy (SEM) images and EDS analyses were obtained on Gemini 300 (Zeiss). FT-IR spectra were recorded as described above. Surface compositions of mZIF-8/PLA membrane were analyzed by X-ray photoelectron spectroscopy (XPS) (Thermo K-Alpha XPS). The hydrophobicity of the membranes was measured by LSA100 contact angle goniometer (LAUDA Scientific). ZETA system (SurPASS) was employed for analyzing the zeta potential of the membranes before and after mineralization.

### Cellular responses in vitro

Cell extraction and cell culture: This study involved the separation and collection of BMSCs using the following procedure. Neonate Sprague Dawley (SD) rats aged 3–5 days were euthanized by cervical dislocation, followed by a 30-minute immersion in 75% alcohol. The muscles and soft tissues attached to the femurs and tibias were then removed, and the cartilages at the two ends of the bones were cut. The cavities were repeatedly rinsed with culture medium until they turned white. Subsequently, the freshly collected bone marrow was inoculated into 10-cm culture dishes containing culture medium. The dishes were then incubated at 37 ℃ with 5% CO_2_ (Heracell150i, Thermo Scientific), with the medium being changed every 3 days. Once the BMSCs reached confluency, cell passage was performed. Every in vitro experiment repeated at least 6 times with *n* = 6 technical replicates (six-well plate).

Biocompatibility evaluation: A Cell Counting Kit-8 (CCK-8) test (Dojindo) was conducted to assess the cell growth and cytotoxic activity of the membranes. The membranes underwent a 7-day pre-mineralization in SBF, followed by immersion in a culture medium and overnight incubation. Subsequently, 5000 BMSCs were grown using the membrane leaching solution during the cytotoxic activity test, and BMSCs cells (1.8 × 10^5^) were placed on the membranes during the cell growth test. Calcein AM (live)/PI (dead) was used to determine cell viability for live/dead staining. A seeding density of 2 × 10^5^/well was used for seeding onto the membrane surface. Plates containing membranes were later subjected to 3/7-day incubation under 37℃ with 5% CO_2_ prior to imaging with LSCM (A1R, Nikon).

Osteogenic differentiation: The osteoinductive medium was replenished at 2-day intervals. At 5- and 14-day after cell culture, the qRT–PCR and western blot were performed to analyze osteogenic gene levels and osteogenic protein levels within BMSCs. After 14-d incubation, phalloidin staining for F-actin, osteocalcin (OCN) staining, and DAPI nuclear staining were used. Alizarin Red S (ARS) staining was used to analyze extracellular matrix (ECM) mineralization in cells cultured with leaching solution and those on the membranes. Alkaline phosphatase (ALP) staining was carried out with an ALP Stain Kit (Beyotime), while collagen content was measured using a collagen quantitation kit (Nanjing Jiancheng Corp) following the manufacturer’s instructions.

Polarization of macrophages: RAW264.7 with a seeding density of 2.5 × 10^4^ cells/ml were seeded on the mPLA and mZIF-8/PLA membranes. After 2 d, RAW264.7 were stained with CD86 and CD206 specific antibodies (eBioscience) detected by using a flow cytometer (Beckmam). TNF𝛼, IL6, and IL10 was detected by ELISA kits (ThermoFisher Scientific). For immunofluorescence staining, the macrophages were stained with TRITC-labeled anti-CD86 antibodies, and FITC-labeled anti-CD206 antibodies. The results were obtained from Confocal laser scanning microscopy (CLSM). The qRT–PCR was performed to identify the changes in gene expression related to macrophage polarization (C-C chemokine receptor type 7 (CCR7), CD206, and vascular endothelial growth factor (VEGF)).

### In vivo animal responses to mPLA and mZIF-8/PLA membrane

Bone Defect Reconstruction in vivo: To evaluate the pro-osteogenesis abilities of mZIF-8/PLA membrane, SD rats (6 weeks old, male, 200–250 g) were randomly assigned to three groups (*n* = 8 rats per group): i). Blank, ii) mPLA (left), and iii) mZIF-8/PLA (right). This study was approved by the Experimental Animal Ethics Committee of Huazhong University of Science and Technology under Ethics approval Number: [2019] IEC(S1154). The animal experiment was conducted in accordance with the Guide for the Care and Use of Laboratory Animals, as well as adhering to the ARRIVE guidelines. Prior to implantation, the membranes were immersed in SBF for 7 days pre-mineralization. Rats were anesthetized with 3% w/v pentobarbital sodium, and a 2.5-cm sagittal incision was made in the middle of the skin overlying the rat skull. Subsequently, symmetrical critical cranial defects were drilled using a a circular drill (Goldach).

Micro-CT analysis: Rats from each group were sacrificed using an excess of pentobarbital sodium at 4/12- weeks post-operation and their skull samples were harvested and fixed in 10% formalin for 48 h. New bone formation in the defect areas was evaluated using microcomputed tomography (micro-CT) scanning equipment. All skull samples were scanned at a source of 226 mean threshold value, 9 μm resolution ratio, and circular region of 5.0 mm in diameter and 2.0 mm in depth was chosen as the volume of interest (VOI). VG Studio and CTAn software were used for reconstruction and quantitative morphometric analyses.

Histological and immunohistochemical (IHC) analyses: Sample decalcification in EDTA was conducted for 4 weeks, followed by paraffin embedding, dehydration with gradient ethanol, and slicing in 3-µm sections for H&E staining (Beyotime) and Masson’s trichrome staining. The expressions of OCN and CD31 were evaluated through IHC analysis with specific antibodies.

### Data processing

The test results were graphed using Origin and GraphPad Prism 9.0 software. The obtained data are expressed as mean ± SD. The comparison of means between two groups was verified using the t-test (Student’s t-test). The comparison of means between multiple groups was verified using one-way analysis of variance (One-way ANOVA). If *P* < 0.05, it indicates that the hypothesis test is invalid and there is a significant difference in the means between groups.

## Results and discussion

**Fig. 1:**
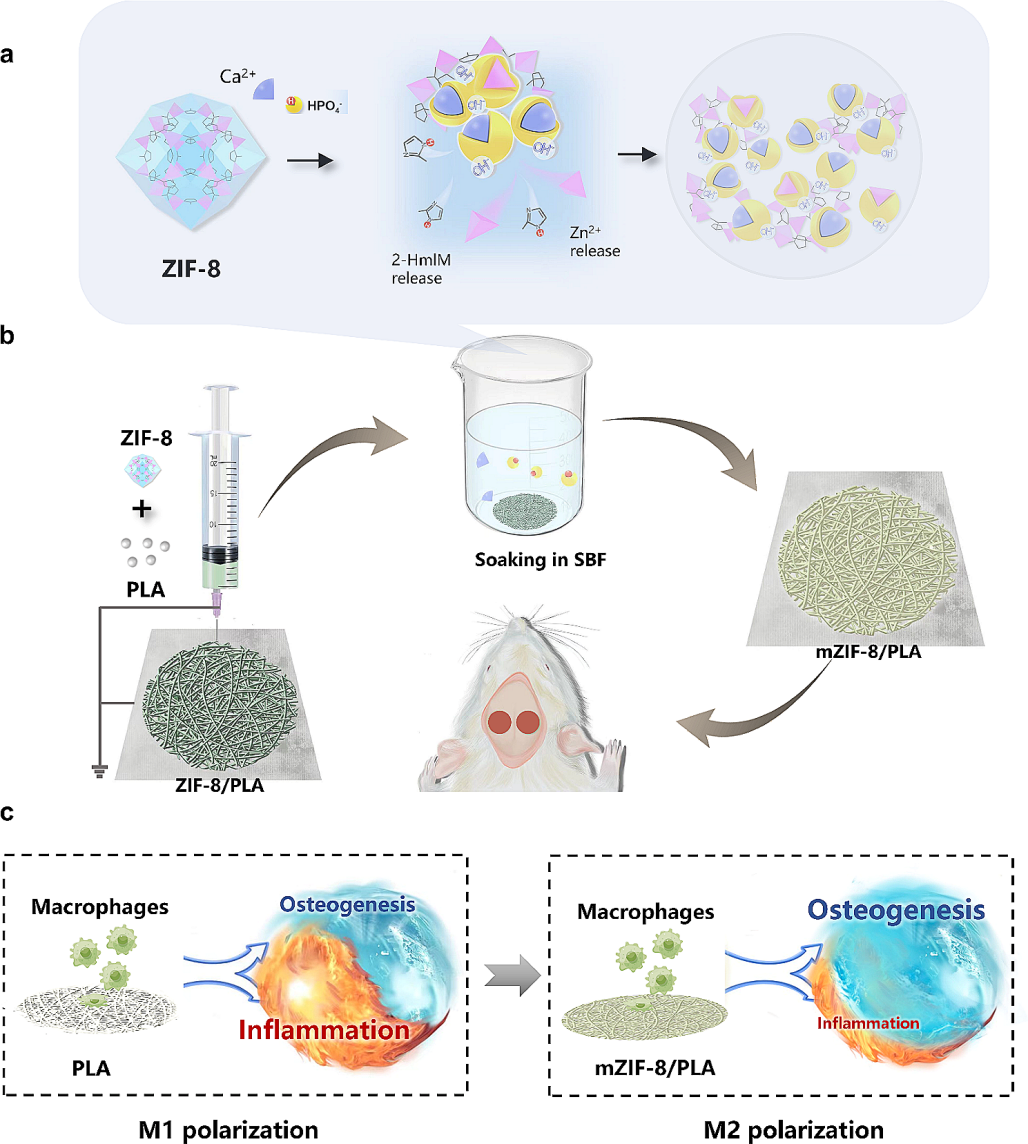
Schematic of spontaneously induced biomimetic mineralization of electrospun ZIF-8/PLA membrane enhanced in situ bone defect repair. **(a)** Mineralization procedure of ZIF-8 in the body fluid microenvironment. **(b)** mZIF-8/PLA membrane with biomimetic mineralization capability, manufactured using electrospinning technology, was implanted into rat critical-sized skull defect models for in vivo bone regeneration evaluation. **(c)** Molecular mechanism of M2 polarization induced by the mZIF-8/PLA membrane

The method framework, as shown in Fig. 1, involved the preparation o f a mZIF-8/PLA membrane using electrospinning technology and soaking it in simulated body fluid. In vitro experiments confirmed the membrane’s ability to induce biomimetic mineralization and regulate bone immunity in the microenvironment of body fluids. Subsequently, the membrane was implanted in rats to assess bone regeneration in a critical-size skull defect model.

### Self-inducing biomimetic apatite deposition by ZIF-8 disintegration process

**Fig. 2:**
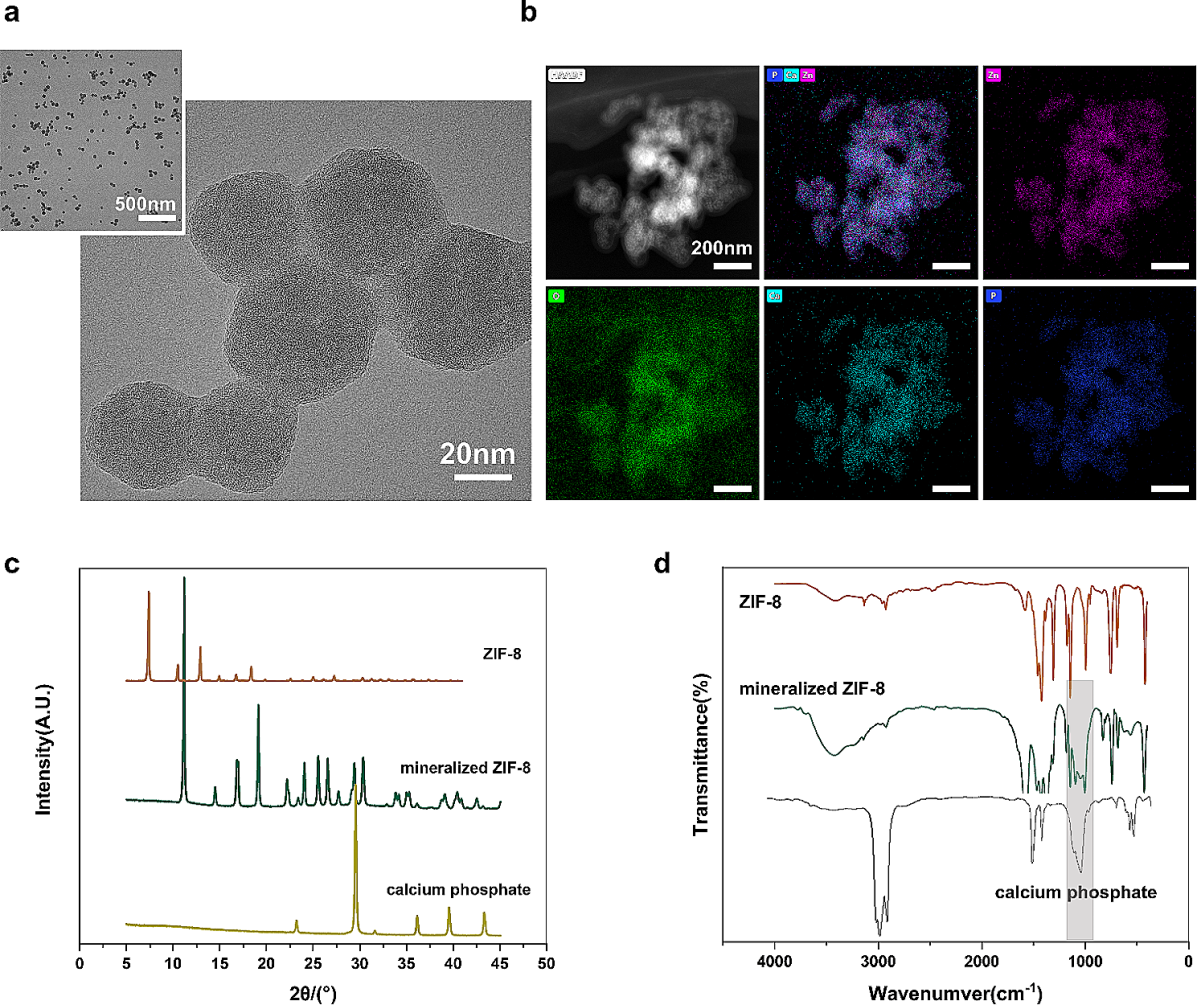
ZIF-8 induces biomimetic mineralization spontaneously. **(a)** TEM images of a typical structure of synthesized ZIF-8 particles. **(b)** EDS elements mapping showing morphology and component changes of ZIF-8 during mineralization process. **(c)** Comparison of XRD patterns of synthesized ZIF-8 and mineralized ZIF-8 (mineralization for 14 days). **d** FT-IR analysis of the original ZIF-8 and mineralized ZIF-8, together with standard phosphate absorption bands

ZIF-8 synthesized using the one-pot method exhibited a homogeneous, rhombic dodecahedral structure, with an average particle size of approximately 50 nm **(**Fig. 2a**)**. After incubation in SBF solution for 14 days, the structure of the prepared ZIF-8 nanoparticles transformed into spherical agglomerates. Further EDS mapping together with TEM images revealed that the increase in volume was primarily due to the deposition of Ca and P-containing substances compared to the original ZIF-8 composition **(**Fig. 2b**).** The powder XRD patterns of the ZIF-8 samples before and after immersion in SBF are presented in Fig. 2c. Comparison of the powder XRD patterns of ZIF-8 samples at different time points during the mineralization process, showed changes in peak intensity from the standard ZIF-8, indicating a possible defective crystal structure and the formation of new composite. FT-IR testing was utilized to discern the differences between mineralized and non-mineralized composite **(**Fig. 2d**)**. The spectra exhibited a gradual decline in the peak intensities of vibration modes related to νC = N (1584 cm^–1^) and νring (1500–1350 cm^–1^). Additionally, the broadbands changes between 1150 and 900 cm^–1^ and 660–530 cm^–1^ may be attributed to the antisymmetric stretching modes and the bending of PO_4_^3−^ groups.

Owing to its exceptional thermal stability, pH responsiveness, and excellent drug loading efficiency, ZIF-8 shows great promise as a material for bone regeneration. Studies have demonstrated its ability to promote osteogenesis through the release of Zn^2+^ ions both in vitro and in vivo [29]. During the degradation process of ZIF-8 in phosphate buffer solution, the coordination equilibrium between Zn^2+^ ions and 2-methylimidazole (2-HmIM) ligand changed. The high affinity of such phosphate groups for polyvalent cation causes the shifting the equilibrium toward forming insoluble inorganic byproducts [17]. This information led to the hypothesis that the appearance of new bands is linked to biomimetic apatite deposition and the reduced formation of zinc phosphates as degradation byproducts of ZIF-8. This indicates that the spontaneous mineralization occurs during the degradation of ZIF-8.

### Fabrication and characterization of electrospun mZIF-8/PLA membrane

**Fig. 3:**
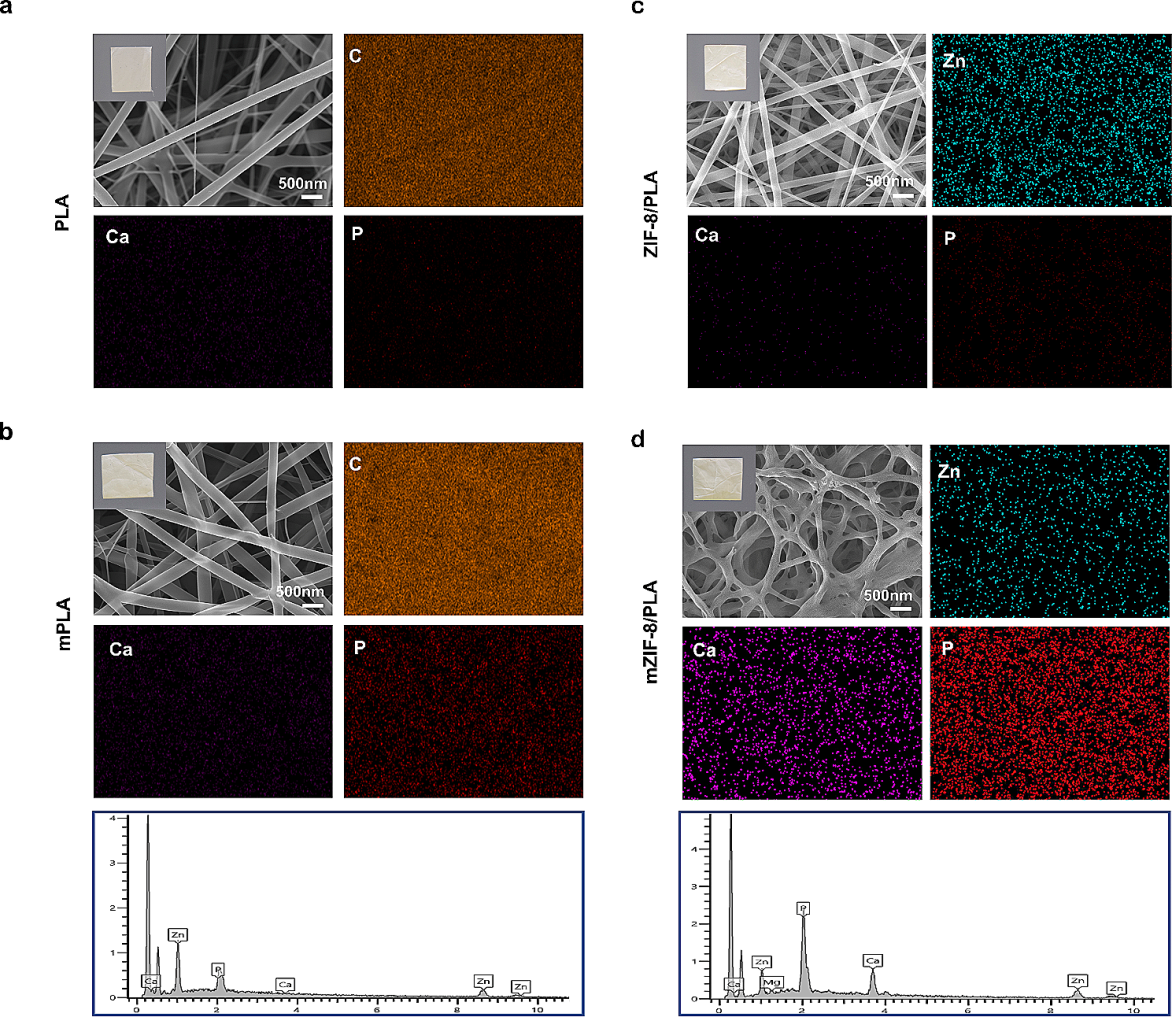
Characterization of the mineralization properties of the mZIF-8/PLA membrane. **a, b)** No significant changes between the PLA and mPLA membrane groups under SEM observation. **c, d)** SEM images of obvious crystal deposition after the mineralization process on the surface of ZIF-8/PLA membrane. Elemental mapping confirms the deposition involves Ca, P, and Zn elements

Following the identification of potential biomimetic mineralization mechanisms in ZIF-8, we proceeded to fabricate PLA and ZIF-8/PLA membrane using electrospinning technology. After 7 days mineralization in SBF solution, SEM images revealed crystal deposits on mZIF-8/PLA **(**Fig. 3c, d**)** compared with the mineralized PLA (mPLA) group with no significant changes **(**Fig. 3a, b**)**, which indicate that PLA fiber diameters remained unchanged with additional ZIF-8 incorporation.

**Fig. 4:**
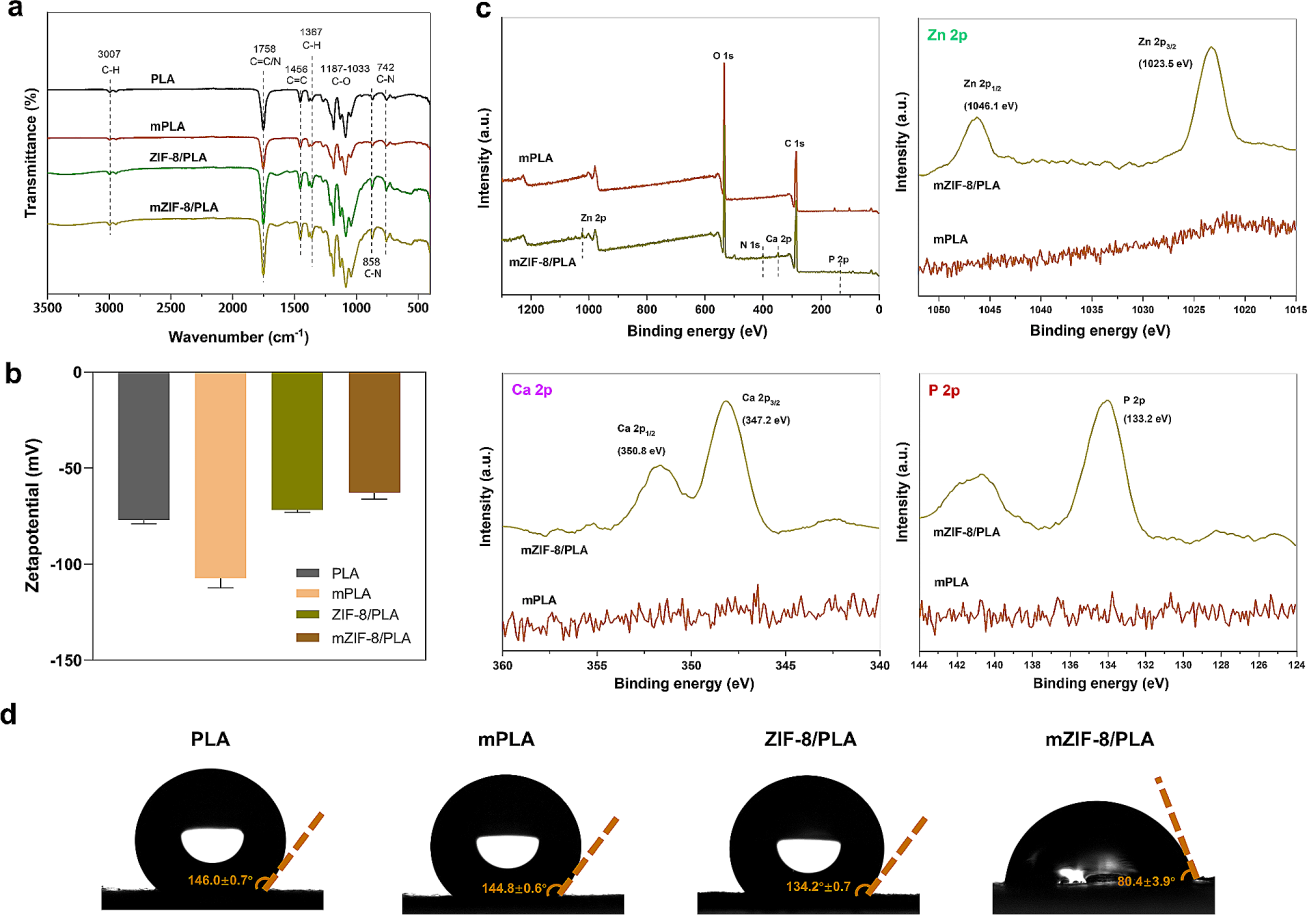
Characterizations of the material properties of the mZIF-8/PLA membrane. **(a)** Comparative FT-IR spectroscopic analysis of the PLA and ZIF-8/PLA membranes after immersion in SBF. **(b)** Zeta potential (ξ) measurements of the dispersion surface charge of the membranes can be altered by adding ZIF-8 particles. **(c)** Surface elemental compositions were determined using XPS. **(d)** Water contact angle test demonstrated that the hydrophily of PLA membrane after 7-day mineralization significantly increased due to the addition of ZIF-8

EDS elements mapping confirmed the presence of calcium, phosphorus, and zinc elements on the surface depositions. FT-IR spectra of PLA and ZIF-8/PLA immersed in SBF revealed the structural information of the mixture, including the bands of C–N, C = C/N, C–H, and C–O **(**Fig. 4a**)**. No significant differences were observed between the membranes post-treatment. The surface charge of the particles was determined by measuring zeta potential **(**Fig. 4b**)**. After biomineralization, we observed a higher negative zeta potential of mPLA and a lower negative zeta potential of mZIF-8/PLA. The XPS analyses showed that zinc, nitrogen, phosphorus, and calcium were doped in mZIF-8/PLA **(**Fig. 4c). The aforementioned results could prove that the PLA membranes containing ZIF-8 could form a calcium–phosphorus layer and that PLA had no calcium–phosphorus layer formation. Additionally, the modification of surface properties due to the presence of ZIF-8 and its biomimetic mineralization capacity was confirmed through contact angle measurements, showing a significant decrease in water contact angle values **(**Fig. 4d**)**.

The surface composition of electrospun membranes is a critical factor in their response and integration, as supported by previous research [30]. PLA is a widely used biocompatible polymer material with satisfactory toughness and negligible cytotoxicity. However, PLA-only electrospun membranes have no functional groups on the surface and lack of recognition sites for osseointegration, prolonging bone-healing time [31]. ZIF-8 nanoparticles can be compounded into fibrous membranes with PLA well by the electrospinning technology, showcasing a biomimetic mineralization feature on the composite membrane. Zeta potential, one of the key properties of materials, can affect cell adhesion [32]. A high absolute value of zeta potential can increase the repulsive force between materials and cells and reduce their adhesion [33]. The lowest zeta potential of mZIF-8/PLA indicated better cell adhesion potential because of the mineralization effect. Furthermore, the higher hydrophilicity may facilitate osteoblast growth.

### Biocompatibility of mZIF-8/PLA membranes in vitro

**Fig. 5:**
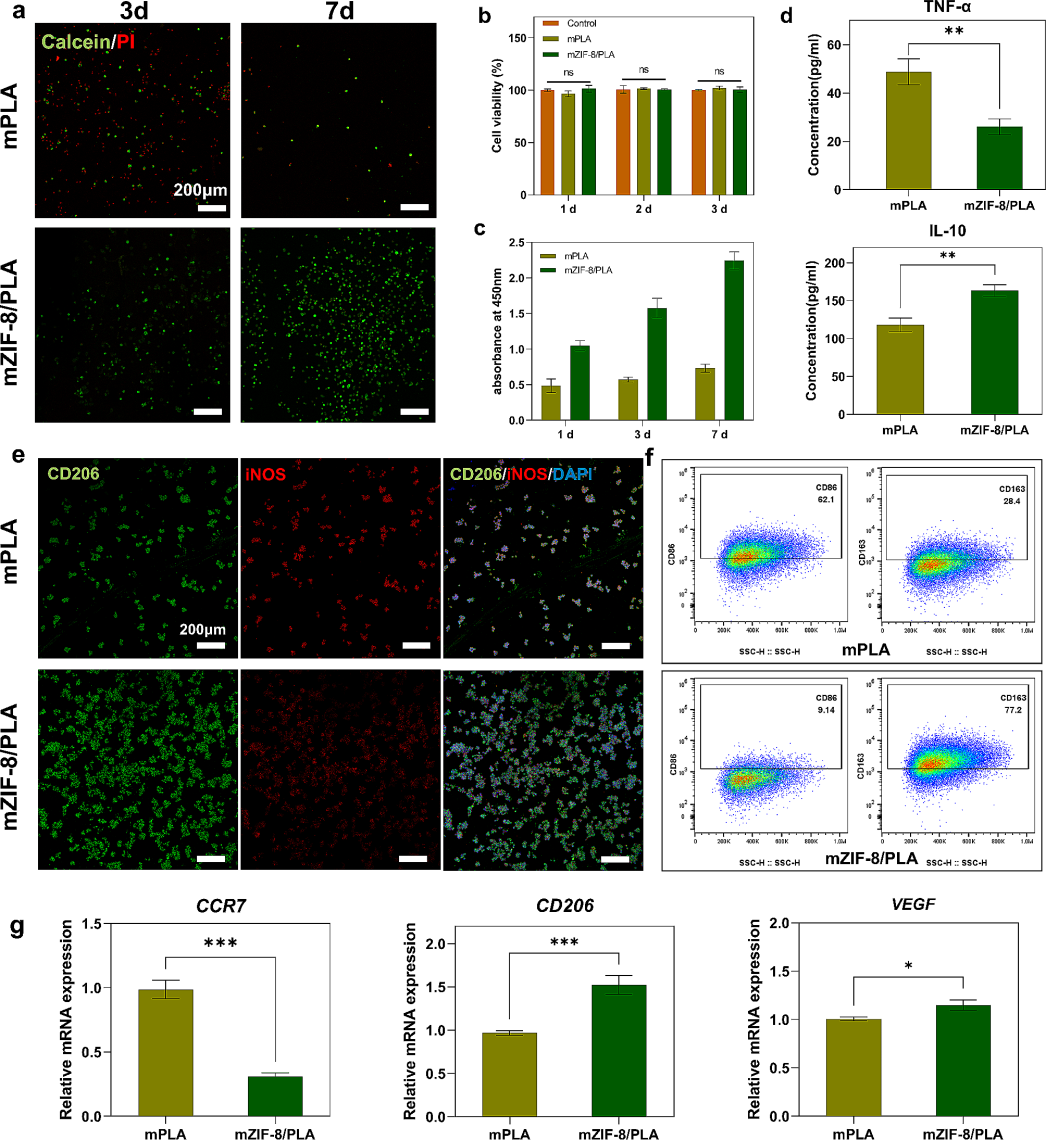
Biocompatibility for BMSCs and the phenotype of macrophages cultured on the mZIF-8/PLA membrane. **(a)** Immunofluorescent staining for Calcein AM (green) and PI (red) in BMSCs from mPLA and mZIF-8/PLA group at day 3 and day 7. **(b)** Cell cytotoxicity assessment of BMSCs co-cultured in mPLA and mZIF-8/PLA groups showed no significant differences compared to the control group. **(c)** Cell viability result of BMSCs co-cultured with mZIF-8/PLA membrane shows a higher proliferating ability. **(d)** TNF𝛼 and IL10 production of 2 day–cultured macrophages by ELISA. **(e)** Surface markers IF staining and quantitative analysis of seeded macrophage (inducible nitric oxide synthase (iNOS) and CD206). **(f)** Flow cytometry demonstrating the percentages of CD86-positive and CD163-positive macrophages after cell seeding for 2 days. **(g)** CCR7, CD206, and VEGF of macrophages evaluated by RT–PCR. *p < 0.05, **p < 0.01, *** *p* < 0.001 (*n* = 6)

The material characteristics were determined and the adhesion and cell viability of bone marrow mesenchymal stem cells (BMSCs) were evaluated for each group. BMSCs were seeded on the mPLA and mZIF-8/PLA groups, and Calcein AM and potassium iodide were further stained at specified times for CLSM imaging **(**Fig. 5a**)**. BMSCs on the mZIF-8/PLA membrane exhibited strong adhesion, rapid growth, and even distribution, whereas the cells on the mPLA group showed minimal adhesion and proliferation. CCK8 cell proliferation assays further confirmed this result **(**Fig. 5b**)**. In vitro cytotoxicity test of mZIF-8/PLA membrane was also evaluated using CCK8 kit. As shown in Fig. 5c, neither of the membranes significantly affected the viability of BMSCs. Therefore, the CCK8 assay and live/dead staining results indicated that mZIF-8/PLA membrane demonstrated satisfactory cytocompatibility, serving as the foundation for subsequent experiments.

### Evaluation of macrophage polarization

To further evaluate the macrophage polarization, RAW264.7 cells were seeded on mPLA and mZIF-8/PLA individually. The M1 phenotype, known for high CD86 levels and low CD206 levels along with increased secretion of TNF-α, iNOS, and interleukin-6 (IL-6), was compared to the M2 macrophages characterized by higher IL-10 and IL-4 secretion, and increased CD163 and CD206 expression. ELISA was applied to concentration detection of TNF-𝛼 and IL10 after culturing for 2 days to assessing macrophage polarization (Fig. 5d). the secretion of TNF-𝛼 was significantly inhibited in the mZIF-8/PLA group. These results indicated that the mZIF-8/PLA group could can hinder the M1 polarization and related cytokine secretion while facilitating the generation of the M2 phenotype which is beneficial for osteogenesis. Additionally, IF staining revealed an increase in iNOS-positive cells on the mPLA membrane and more CD206-positive cells on mZIF-8/PLA membrane **(**Fig. 5e**)**.

The gene expression levels of macrophage were analyzed using qRT–PCR. The expression of CD206 significantly increased, while expression levels of CCR7 notably decreased in the mZIF-8/PLA group. Moreover, mZIF-8/PLA upregulates the expression of VEGF and thus angiogenesis and related M2 polarization (Fig. 5g). Further, FCA was performed to further confirm the results (Fig. 5f). CD86-positive cells in mPLA group (62.1%) were more present than mZIF-8/PLA group (9.14%). Conversely, CD163-positive cells were more present in mZIF-8/PLA group (77.2%) compared to mPLA group (28.4%). These results indicate that mZIF-8/PLA tends to induce macrophages polarization towards the M2 phenotype.

Ca–Zn–P chemical conversion coating and biomimetic calcium phosphate coatings were proven to exert osteoimmunomodulation effects by releasing ions (through PI3K/Akt and Wnt pathways) and changing morphology of materials surface (through Rho-GTPase pathways) to act as possible mechanisms of M2 polarization 27.^34^. Although pure ZIF-8 did not significantly affect the polarization of macrophages [35], the mineralized ZIF-8 exhibited a biological effect similar to that of the above coating structure. This can also be attributed to the spontaneously induced calcium–phosphorus deposition on ZIF-8.

Research in recent years has investigated the impact of various metal elements on immune regulation. According to some reports, degradation products of Zn-based implants have been found to diffuse effectively into the surrounding tissue the implant and prevent osteolysis [36]. The potential antibacterial mechanisms of zinc-based implants may include inhibiting biofilm formation, activating autolysis-related pathways, and antibiotic resistance. Additionally, zinc-based implants may benefit osseointegration by inhibiting the expression of osteoclast-related proteins, without affecting the expression of osteogenesis-related proteins, and without activating the expression of related inflammatory proteins [36]. Liu et al. [37] discovered that coating SPEEK biomaterials with a layer of zinc ions, forming a zinc coating, can regulate the microenvironment of SPEEK, thereby influencing the polarization of inactivated M0 macrophages to M1 anti-inflammatory phenotype. This zinc coating also induces and promotes the secretion of anti-inflammatory factors and osteogenesis-related cytokines, ultimately improving the osseointegration between zinc-coated SPEEK and bone tissue. In our study, Zn^2+^ can be released from ZIF-8 during the biomimetic mineralization process, playing a role in the local microenvironment.

Also, adding Ca^2+^ into the local microenvironment could enhance the physical and chemical properties of the implant and its ability to modulate the immune response [25]. On one hand, Ca^2+^ can promote the polarization of macrophages towards M2 through the Wnt/𝛽 and PI3K-Akt signaling pathways [38]. On the other hand, Ca^2+^ signaling is involved in osteoclast differentiation and bone resorption. The oscillatory changes in intracellular Ca^2+^ concentration, induced by activation of the RANKL signaling pathway, play a crucial role in osteoclast differentiation. These changes stimulate osteoclast specificity by activating the nuclear factor of activated T cells and the nuclear factor of activated T-cells 1 (NFATc1) pathway in the cytoplasm. It is important to note that osteoclast differentiation relies not only on the release of intracellular Ca^2+^, but also on the influx of extracellular Ca^2+^ [40]. Additionally, studies have shown that Ca–Zn–P chemical conversion coating and biomimetic calcium phosphate coatings were proven to exert osteoimmunomodulation effects by releasing ions (through PI3K/Akt and Wnt pathways) and changing morphology of materials surface (through Rho-GTPase pathways) to act as possible mechanisms of M2 polarization [27, 34]. 

### In vitro evaluation of BMSCs behavior

**Fig. 6:**
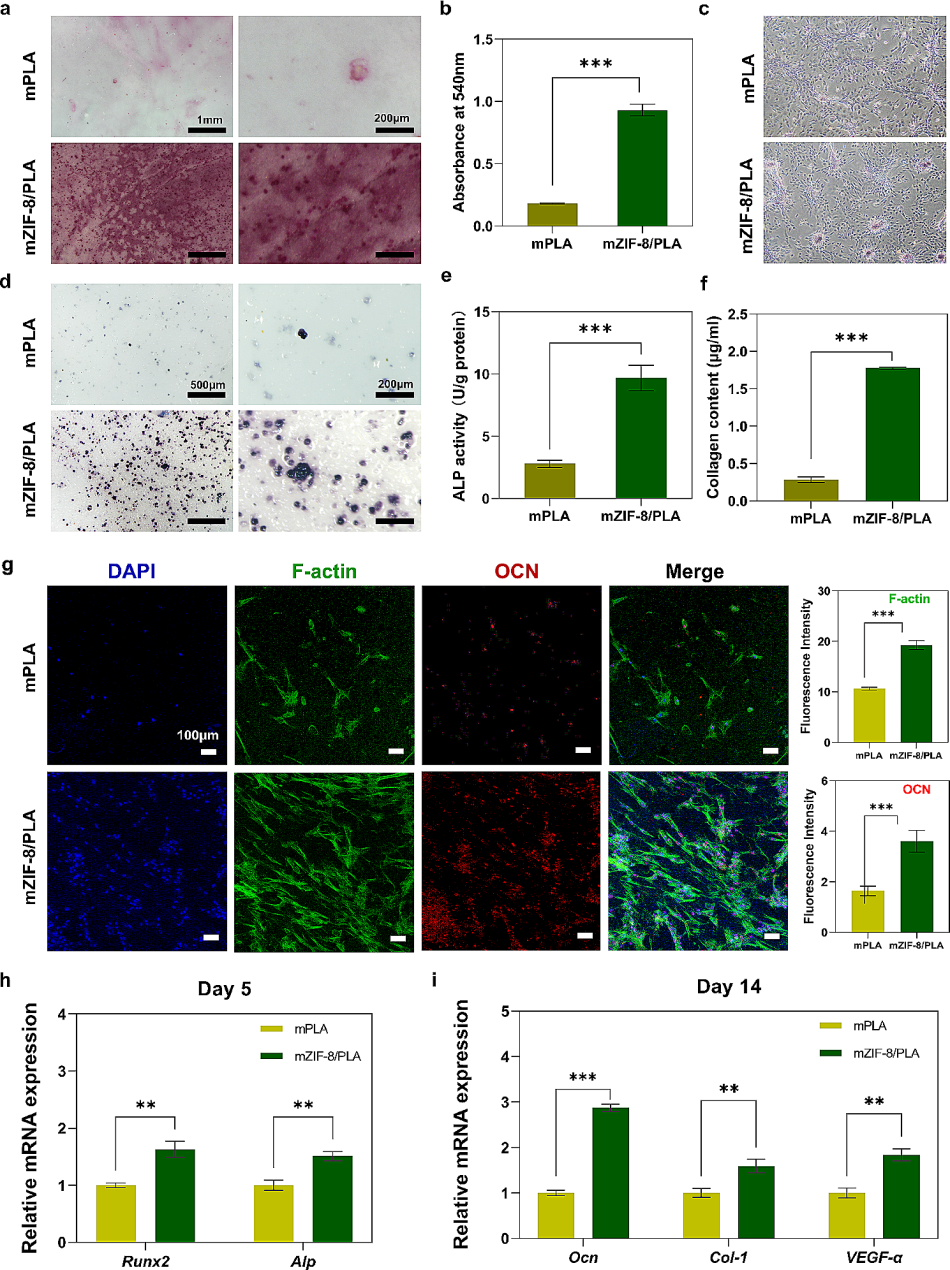
Pro-osteogenesis ability of BMSCs co-cultured with various membranes in vitro. **(a)** View of mPLA and mZIF-8/PLA after culturing with BMSCs in osteogenic induction medium in Alizarin Red staining at day 14. **(b)** Alizarin Red semi-quantification of calcium deposition to represent mineral deposition. **(c)** The mineralization ECM of BMSCs incubated with leaching solutions from different groups in Alizarin Red staining. **d, e)** ALP staining and quantification of ALP activity. **f)** Collagen content was estimated by determining the hydroxyproline content. **g)** Fluorescent images and quantitative analysis of OCN expression after 14 days of culture; Red (OCN), green (F-actin), and blue (nucleus). **h)** In the mZIF-8/PLA group, gene expression of *Runx2* and *Alp* in BMSCs at day 5, *Ocn*, *Col-1* and *vegf* in BMSCs at day 14, were significantly increased compared with the mPLA group. **p < 0.01, ***p < 0.001 (*n* = 6)

In order to assess the ability for bone regeneration of mZIF-8/PLA membranes in vitro, it is important to examine its ability to support cell proliferation and differentiation [39]. To determine the potential of the GBR membranes in promoting new bone growth, the osteogenic differentiation and cell viability of BMSCs was analyzed. We supplemented the cell culture medium with osteogenic induction components for better observation. ECM calcium deposits on the membranes were showed by alizarin red staining. Compared with control cells, the BMSCs seeded on the mZIF-8/PLA membranes exhibited enhanced mineralization on day 14 **(**Fig. 6a**)**. This was also confirmed by the quantitative analysis **(**Fig. 6b**)**. To determine the effect of zinc ions and other elements releasing from mZIF-8/PLA membranes on osteogenic differentiation, we incubated with BMSCs a leaching solution of membranes **(**Fig. 6c**)**. We also got better results with mZIF-8/PLA group than mPLA group.

ALP is critical to the original stage of bone mineralization process and is regarded an early biomarker of osteogenic phenotype [40]. The ALP activity of BMSCs was examined with ALP detection kit **(**Fig. 6d**)**, which revealed that the dye-labeling density of the mZIF-8/PLA membrane was significantly higher compared with the mPLA membrane. Quantitative results also presented the similar trend. **(**Fig. 6e**)**, indicating that the mineralization of ZIF-8 enhanced early osteogenic differentiation of BMSCs to a larger extent. Because hydroxyproline is primarily restricted to collagen, the determination of hydroxyproline content can be used to indicate the collagen content in a tissue. Total collagen content was measured through the determination of hydroxyproline concentration [41]. We found from the results that the collagen production by BMSCs cultured on mZIF-8/PLA was significantly more than that on mPLA **(**Fig. 6f**)**.

The synthesis of Osteocalcin (OCN), a protein related to the late period of osteogenic differentiation, plays a crucial role in modulating matrix mineralization and new bone formation [42]. As shown in Fig. 6g, the expression of OCN within BMSCs was visualized through immunofluorescence staining images after 21 days of culture. Compared with mPLA, the red fluorescence intensity on mZIF-8/PLA was stronger. Moreover, quantitative analysis revealed that the OCN expressed in the BMSCs seeded on mZIF-8/PLA was approximately 2.5 times versus mPLA. Expressions of osteogenesis-related genes were estimated via qRT–PCR. The mZIF-8/PLA group exhibited significantly higher levels of *ALP, RUNX2*, and *OCN*, which increased by 1.6-, 1.5-, and 2.8-fold, respectively **(**Fig. 6h, i**)**. Besides, expressions of *COL-1* and *vegf-α* were also increased in the mZIF-8/PLA group by 1.6- and 1.8-folds, respectively, which might be considered an effective clue for future research to explore the pro-angiogenic capacity.

In this study, mZIF-8/PLA membrane demonstrated favorable cellular responses in terms of cell attachment, adhesion, and proliferation. The cell activity observed in the mZIF-8/PLA membrane can be attributed to multiple factors. During the electrospinning process, both the surface of ZIF-8 particles and the electrospun PLA fibers carry a negative charge. As a result, the surface of ZIF-8/PLA tends to be negatively charged, which facilitates the nucleation of hydroxyapatite crystals [43]. ECM molecules responsible for cell adhesion also possess a negative charge, making them more likely to be attracted to positively charged surfaces. Therefore, positively charged surfaces are generally considered to have better cell adhesion than negatively charged surfaces [44–46]. The research findings indicate that a positively charged calcium and phosphorus deposition layer forms during the mineralization process of ZIF-8/PLA. The zeta potential of ZIF-8 particles decreases, gradually transforming the surface into a positively charged one. Consequently, the presence of the calcium-phosphorus deposition layer enhances the cell adhesion of the material surface. Furthermore, the mZIF-8/PLA membrane prepared using electrospinning technology significantly influences the osteogenic differentiation of co-cultured BMSCs.

Based on the aforementioned experimental results, it can be confirmed that ZIF-8 composite polymer materials offer several significant advantages for bone regeneration research. Firstly, the ZIF-8/PLA membrane has the ability to spontaneously induce mineralization and crystallization in a body fluid environment sedimentary layer. Secondly, the material forms a rough fiber surface structure, which has been shown in previous reports to enhance the initial adhesion of BMSCs [47]. Lastly, the release of Zn^2+^ is another beneficial aspect of ZIF-8 composite polymer materials. The role of zinc in bone metabolism has been supported by various cellular and molecular evidence, including its ability to enhance osteoblast proliferation, ALP activity, and influence the synthesis of osteopontin and osteocalcin at the protein level [48]. Additionally, zinc can activate the extracellular signal-regulated kinase pathway, leading to changes in the expression levels of osteogenesis-related genes like Runx2 [49]. In the case of the mZIF-8/PLA membrane, the gradual exposure of ZIF-8 during the PLA fiber degradation process promotes both zinc release and biomimetic mineralization initiation, thus contributing to bone differentiation. However, further research is required to fully understand the details of this dynamic process.

### New bone formation in vivo

**Fig. 7:**
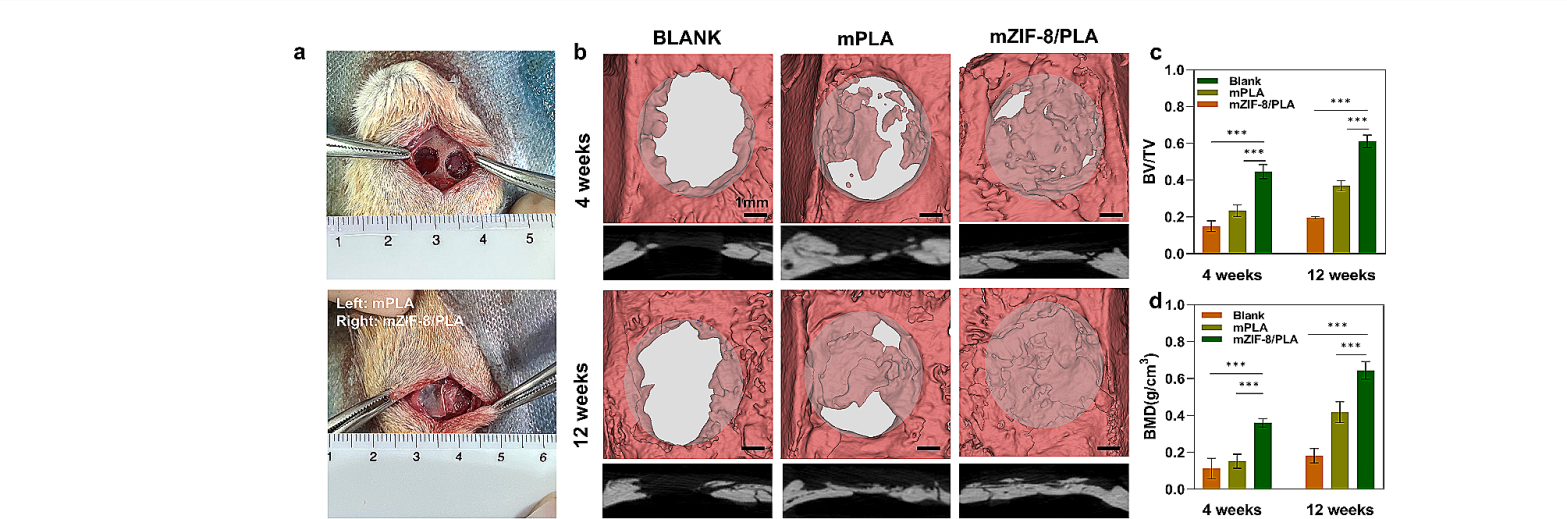
New bone formation *in vivo.***a)** Macroscopic observation of implantation area intraoperatively. **b)** 3D reconstructed images after micro-CT scanning of the calvarial defect area. Quantitative results of **c)** BV/TV and **d)** BMD. ***p < 0.001 (*n* = 8)

After demonstrating the osteogenic effect of mZIF-8/PLA membranes in vitro, we evaluated the in vivo osteogenic bioactivity in a rat calvarial defect model **(**Fig. 7a**)**. Rats were sacrificed at 4/12-weeks after surgery, and bone reparation was assessed by micro-CT and histological analysis. Reconstructed micro-CT images show newly formed bone within the defect area **(**Fig. 7b**)**.

The results showed better new bone formations in all membranes-implant groups, with the highest new bone formation in the mZIF-8/PLA membrane group. Moreover, mZIF-8/PLA membrane group showed substantial bone ingrowth centripetally from the original defect edge toward the defect center, as well as in the center area. Quantitative analysis of basic parameters such as BV/TV and BMD showed in Fig. 7c and d. The mZIF-8/PLA group induced a higher growth kinetics of new bone formation and improved average bone density, compared to the primitive mPLA membrane group.

**Fig. 8:**
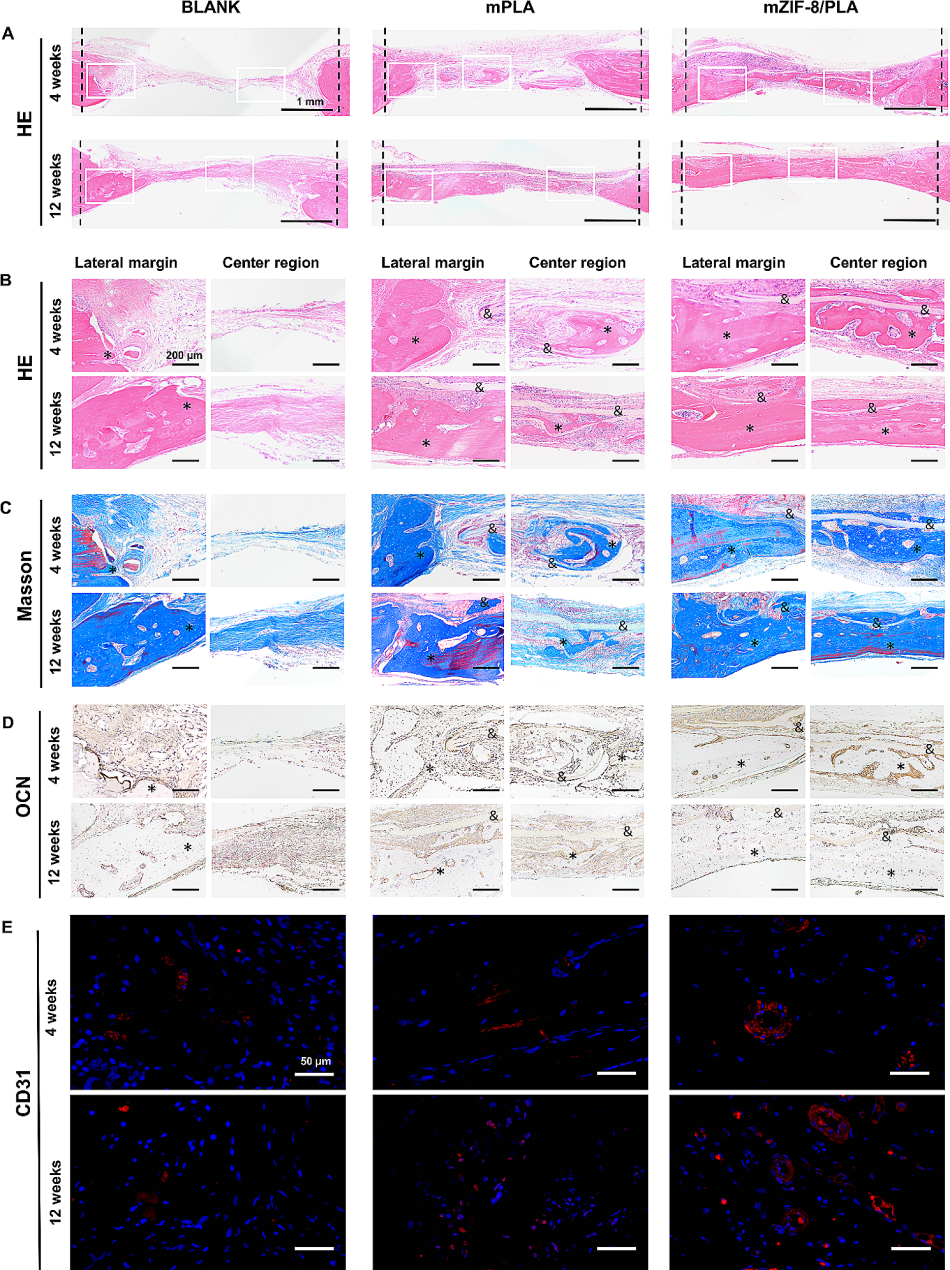
Histological and immunohistochemical analysis. **(a)** H&E staining (the margin and the center region of the defects). **(b–e)** H&E staining **(b)**, Masson’s trichrome staining **(c)**, OCN staining (D), and CD31 staining **(e)** images of the mPLA and mZIF-8/PLA bone guide membranes (*: new bone, &: membranes)

Micro-CT findings were confirmed by histological evaluation based on H&E **(**Fig. 8a, b**)**, Masson’s trichrome **(**Fig. 8c**)**, and immunohistochemical staining **(**Fig. 8d**)** of demineralized bone specimens. In the mZIF-8/PLA group, neonatal bone appears at the edge of the defect as well as in the central region after 4 weeks of implantation, indicated osteoconductive abilities of the mZIF-8/PLA membrane. Even more surprising, the defects were almost entirely closed by 12 weeks after surgery. Immuno-histochemical staining slides showed that mZIF-8/PLA group exhibited the maximal OCN-positive expression area.

In support of the hypothesis that ZIF-8 loading positively affects angiogenesis, we stained by immunofluorescence for CD31, a specific protein involved in vascularization (Fig. 8e). Throughout the repair process, compared with the other groups, there was more CD31-labeled neovascularization in mZIF-8/PLA groups, indicating that mZIF-8/PLA membrane was beneficial to the angiogenesis within new bones. The enhanced angiogenesis was attributed to the activation of calcium-sensing receptors on endothelial progenitor cells by calcium ions, leading to upregulation of VEGF and VEGF receptor 2 expression [51]. Consequently, the in vivo bone repair experiment results support the notion that mZIF-8/PLA holds promise for vascularized bone regeneration.

## Conclusion

The capability of ZIF-8 to spontaneously induce biomimetic mineralization effectively facilitates biocompatibility and hydrophily when combined with an electrospun PLA membrane. Additionally, ZIF-8 exerted the dual functionality in inducing osteogenesis and M2 polarization, promoting the secretion of anti-inflammatory and pro-osteogenic factors. Furthermore, the study findings revealed that the novel GBR membrane revealed a remarkable osteogenic property, causing vascularized bone remodeling and regeneration. In conclusion, the mineralized ZIF-8/PLA membrane shows promise as an implant for bone repair.

## Data Availability

The data that support the findings of this study are available on request from the corresponding author, upon reasonable request.

## Abbreviations

ALP: Alkaline phosphatase
ARS: Alizarin Red S
BMSCs: Bone marrow mesenchymal stem cell
CCR7: C-C chemokine receptor type 7
ECM: Extracellular matrix
GBR: Guided bone regeneration
IHC: Immunohistochemical
IL: Interleukin
mPLA: Mineralized polylactic acid
mZIF-8/PLA: Mineralized zeolitic imidazolate framework-8/polylactic acid
OCN: Osteocalcin
PBS: Phosphate-buffered saline
PLA: Polylactic acid
SBF: Simulated body fluids
SEM: Scanning electron microscopy
TEM: Transmission electron microscopy
VEGF: Vascular endothelial growth factor
VOI: Volume of interest
XPS: X-ray photoelectron spectroscopy
ZIF-8: Zeolitic imidazolate framework-8
